# Social Rank or Social Bonds: Which one Facilitates Coalition Formation in Male Tibetan Macaques?

**DOI:** 10.3390/biology11091269

**Published:** 2022-08-26

**Authors:** Yi-Mei Tai, Meng-Meng Chen, Yu-Heng Zhang, An-Xiang Ma, Huan Wang, Xi Wang

**Affiliations:** 1 School of Resources and Environmental Engineering, Anhui University, Hefei 230601, China; 2 International Collaborative Research Center for Huangshan Biodiversity and Tibetan Macaque Behavioral Ecology, Hefei 230601, China

**Keywords:** social bond, social rank, coalition, social cooperation, male Tibetan macaques

## Abstract

**Simple Summary:**

The social behavior and the evolution of non-human primates have always been a focus of animal behavior. In this study, we determined that male Tibetan macaques (*Macaca thibetana*) formed a strong social bond, and there were two mechanisms (social bond and social rank) to promote the formation of coalitions. This study provides a new perspective for understanding the maintenance mechanism of social stability in non-human primates under competitive environments.

**Abstract:**

Previous studies have shown that fierce competition could promote male coalitions. There are two ways for males to choose their allies in the conflict. The first is that supporters choose high-ranking individuals, and the second is that supporters choose affiliated individuals. It is necessary to clarify the factors for forming a coalition and the process of cooperation in primates with complex relationships and strict hierarchies. Thus, we conducted a study on a group of free-ranging Tibetan macaques (*Macaca thibetana*) in Anhui, China, and recorded the whole process of male macaques forming coalitions once agonistic support occurred. The results showed that a higher intensity of the social bond between males was associated with more frequent coalitions. Dominance rank also significantly influenced male coalitions, showing that high-ranking individuals allied more frequently. Moreover, males with longer residence times formed more stable coalitions. We suggest that male Tibetan macaques form a stable social relationship, where social bond and social rank both promote the formation of a coalition. This study provided some insights into the mechanism by which social cooperation develops in multi-male and multi-female groups.

## 1. Introduction

Group living in animals developed due to the long-term adaptation to the environment. Compared to a solitary lifestyle, living in groups allows animals to perform various activities, which can greatly enhance information sharing to perform social activities more efficiently, such as collective foraging and avoiding predators [1]. However, social animals often compete for food and space which produces conflicts. Banded mongooses (*Mungos mungo*) are often more aggressive towards intruders or out-groups when food is scarce [2]. Competing for suitable rock substrate, snappers (*Telmatochromis temporalis*) of the larger ecomorph outcompete adults of the smaller, compelling the smaller snappers to use poor shell habitat [3]. Among social animals, competition is particularly obvious in males because they often fight for a mate [4]. For example, long-tailed macaques (*Macaca fascicularis*) may express a preference toward highly valuable females by investing more in aggression and vigilance (investing more time and being more aggressive toward males) while mate-guarding these females [5].

Fierce male-male competition can drive males to form a firm coalition [6]. Males usually increase their competitive advantage and mating success by forming coalitions [7]. In some cases, individuals with similar ranks form coalitions to acquire more resources. In Assamese macaques (*M. assamensis*), males may utilize all-up coalitions (where all aggressors are lower ranking than the target) to attain food resources and higher social rank [8]. In other cases, individuals of different social ranks form coalitions to strengthen social bonds [9]. A social bond is a special kind of social relationship, which may result from repeated dyadic social interactions [10]. Barbary macaques (*M. sylvanus*) choose their allies according to affiliative interactions to reduce the risk of defection in coalitions [11]. In addition to social rank and social bonds, the formation of coalitions may also be influenced by factors such as age and residence time. Young individuals gain benefits through coalitions. In chimpanzees (*Pan troglodytes*), adolescent males form coalitions more often than other members of their group, and thus, have an advantage when competing for ranks as adults [12]. Namely, the social behavior of adolescent males influences the formation of a coalition during adulthood. Additionally, the residence time of different individuals (males of the group and immigrating males at different times) impacts the coalition. Coalitions might help immigrants to integrate into a group more quickly. In Japanese macaques (*M. fuscata*), outgroup males are more likely to engage in social grooming with their partners after immigrating into a new group [13]. We believe that some factors, including social rank, social bonds, age, or residence time might affect the formation of a coalition under different social contexts.

In primates, coalitions can be formed in two ways: (1) Coalitions are formed by supporting high-ranking individuals during conflicts [14]. (2) Coalitions can be formed through social bond strength [15]. Male coalitions in a social group have always been the focus of animal behavior research, but the research on the factors influencing the formation of coalitions is still lacking. More importantly, it is necessary to clarify the factors for forming a coalition and the process of recruiting allies in primates with complex relationships and strict hierarchies.

Tibetan macaques (*M. thibetana*) live in hierarchical social groups. To maintain group benefits and their dominant ranks, male Tibetan macaques need to establish cooperative relationships and form coalitions during particular periods (e.g., mating season) [15]. Previous studies on social relations and cooperative behaviors mostly concentrated on relationships between females or their relatives [16]. To answer whether/how social relations among male individuals affect male-male cooperation, we recorded the whole process of males forming coalitions once agonistic support occurred in Tibetan macaques and predicted that (1) to maintain social rank [17], males with close social bonds would form coalitions more often. (2) To contest fecundity and gain more resource advantages [18], high-ranking males would be more frequently involved in coalition behavior than other-ranking males. (3) Young males would participate in coalition behaviors more frequently than adult males, which in turn gives them an advantage in future competitive dominance [12,19]. (4) The coalition behavior of male immigrants would be more likely to occur than male residents in the group because male immigrants are more likely to be attacked by males within the group [20].

## 2. Materials and Methods

### 2.1. Study Site and Subjects

We studied a habituated group of Tibetan macaques (group Yulinkeng 1, or YA1) inhabiting the “Valley of the Wild Monkeys” (30°04′25.1″ N, 118°08′59.3″ E) at Huangshan, China, at an altitude of 600~1200 m. Due to continuous observations and studies on this group since 1986, all individuals in the group could be accurately identified based on their natural characteristics, and the maternal relationship of all individuals could be determined. In this study, adult and adolescent males from group YA1 (including nine adult and two adolescent males) were selected as the subjects.

### 2.2. Data Collection

During the study period (November 2021 to January 2022), all behavioral data were collected from the group from 08:00 to 17:00. We used the focal animal sampling method and continuous recording method to collect behavioral data, including proximity (5 m). Each focal sampling period was set as 10 min and the order of the focal individual was selected by random drawing [21]. Ad libitum sampling was performed to record the data on aggression-submission and agonistic support behaviors between males. Aggression-submission behaviors include staring, ground slapping, chasing, seizing, biting, avoiding, and fleeing [22,23]. When an attack occurred, the type, initiator, receiver, and third-party supporter of the attack were recorded. The behavioral data were recorded using a Lenovo D66 recorder (Lenovo China, Beijing, China) and a digital video camera (SONY China, Beijing, China). Detailed behavioral definitions are shown in Table 1 [23].

### 2.3. Dominance Hierarchy of Males

We ranked the aggression-submission bouts of adult males to determine their rank order of dominance. The number of wins and losses in conflicts was put in a paired interaction matrix, and the rank order was determined by minimizing the number of wins below the diagonal. We converted these sequential levels into proportional levels, which ranged from 0 (lowest level) to 1 (highest level). Based on the index of aggression-submission behavior and the win-lose ratio, David’s score (DS) was calculated to determine the ranking among individuals [24]. David’s score was calculated using the formula:

$$S=\sum P_{ij}+\sum W_{j}\times P_{ij}-\sum P_{ji}-\sum I_{j}\times P_{ji}$$

Here, $P_{ij}$ represents the ratio of the number of times that individual i defeated individual j to the total number of times that individual i and individual j attacked and submitted; $\;P_{ij}=a_{ij}/n_{ij}$, $a_{ij}\;$ represents the number of times individual i defeated individual j; $n_{ij}$ represents the total number of aggression-submission bouts between individual i and individual j. $\sum W_{j}\times P_{ij}$ represents the $P_{ij}$. weighted sum of the individual i, $W_{j\;}$ represents the sum of all $P_{ij}$ of individual j.$\;\sum P_{ji}\;$represents the sum of all $P_{ji}$ of individual i. $\sum I_{j}\times P_{ji}$ represents the $P_{ji}$ weighted sum of the individual i, $I_{j}$ represents the sum of all $P_{ji}$ of individual j.

We recorded the ranks of adult male Tibetan macaques in the mating season, as shown in Table 2. Based on the aggressive-submission behavior (N = 89, which does not coincide with aggression-submission in agonistic support), we used k-means cluster analysis (100 iterations) to classify each adult male into the following rank classes: high-ranking (YL, YXK, WM), middle-ranking (TQ, NM, DB), and low-ranking (BHZ, DZ, QT).

### 2.4. Male Social Relations

The strength of the social bond between males was measured by determining the individual intimacy index (Dyadic sociality index; or DSI) to evaluate the social relations of males in a group [25]. DSI indicates the degree of deviation of affiliative behavior of a dyad deviates from that of all other dyads in the same group, ranging from 0 to infinity [26]. A high value indicates a strong dyad, while a low value indicates a weak dyad. We calculated the interaction times and duration of social behaviors and recorded two behavioral factors to calculate DSI. These factors included (1) duration of time spent in proximity (second/hour the dyad was observed), (2) number of proximity per h per dyad. The DSI was calculated using the following formula:

$$DSI_{xy}=\frac{\sum_{i}^{d}=1\frac{f_{ixy}}{{\bar{f}}_{i}}}{d}$$

Here, *d* represents the number of behaviors constituting the index (In this study, only proximity data were used, where *d* = 1), $f_{ixy}$ represents the ratio of individual behavior *I* of dyad *x y*, and ${\bar{f}}_{i}$ represents the average ratio of behavior *I* in all dyads social relationships.

### 2.5. Male Coalition Behavior

We used agonistic support among males as a behavioral indicator to quantify coalition behavior. In social animals, agonistic support occurs when an individual participates in an ongoing conflict between two individuals and supports one of them while attacking the other [23]. During agonistic support, the supporter and the recruiter (usually the initiator of the attack) attack the other party (target) simultaneously.

We performed ad libitum sampling to record the data of agonistic support. The identities and behaviors of recruiters, targets, supporters, and bystanders in the attack support events were recorded [27].

When an attack occurs, two attacking individuals are defined as the recruiter and the target. A recruiter was the one who actively sought agonistic support during a conflict (a recruiter, in this case, is an individual chosen by a supporter as an ally). A target was the one who was attacked in a coalition attack. Supporters were individuals who joined one party to attack the other during a conflict. A bystander was an individual who was present within 10 m of the conflict but did not attack.

Additionally, unsuccessful attempts to recruit supporters were also recorded. Recruitment failed when bystanders avoided (avoiding recruitment signals) and ignored (maintaining original behavior) attempts of males to recruit allies.

### 2.6. Statistical Analyses

We performed Spearman’s rank correlation analysis to determine the association between DSI and the formation of the male coalition. We used generalized linear mixed models (GLMMs) with the binomial connection function and the Poisson distribution function to study the factors influencing the coalition and the choice of allies of the supporters (The predictive variables and response variables in the model are shown in Table 3). We used individual ID as a random effect of the models. We conducted all statistical analyses in R version 4.0.5 [28].

Model 1: Would supporters choose the male with the highest ranking as an ally? Whether this choice depends on the strength of social relations?

We used the generalized linear mixed model (GLMM) with the link functions Logit and binomial as distribution functions to determine the factors that influence the selection of partners by supporters. We evaluated whether the ranks of attackers and the DSI scores of supporters and attackers affected the partners selected by the supporters (N = 100). We analyzed each coalition event twice, one of which was whether the supporter and the recruiter were in coalition, and the other was whether the supporter and the target were in coalition. The predictive variables were (1) the rank of the attacker (the high-ranking male individuals in the conflict: yes/no) and (2) the DSI scores of the supporters and the others participating (the recruiter and target) in the conflict. The response variable was whether a male from individuals in conflict was selected as a coalition partner by the supporter (yes/no). To examine the influence of the competitive nature of coalitions we first ran the model including the rank difference between the recruiter and target as an interaction term with the two predictor variables above. If the interaction term was not significant, we then constructed a simplified model that only contained the main effects and analyzed the new model.

Model 2: Was the participation of supporters related to DSI, residence time, or age?

We constructed a generalized linear mixed model (GLMM) with link functions log and Poisson as distribution functions (N = 72). The number of agonistic support events from individual I to J was used as the response variable (“I” is the supporter and “J” is the recruiter). The predictive variables were the residence time/the age of individual I and the DSI scores of individuals I and J. We divided the age of adult males into young adults, middle adults, and old adults (see Table 2).

Model 3: Decision-making of partner choice in coalitions 

We used the generalized linear mixed model (GLMM) with the link function Logit and binomial as distribution functions to evaluate the specific influence of social bonds and social rank interaction terms on coalition formation. We used a dataset containing data on adult male coalition (N = 120). The predictive variable was the interaction of social bond and social rank, and the response variable was the success of the coalition (success: yes/no).

### 2.7. Ethics Statement

The study was completely observational in nature and did not affect the welfare of the Tibetan macaques. This study was conducted with the Huangshan Monkey Management Center and the Huangshan Garden Forest Bureau. It complies with the regulations of the Chinese Wildlife Conservation Association regarding the ethical treatment of research subjects, and under the law of the People’s Republic of China on the protection of wildlife. 

## 3. Results

### 3.1. Male Social Relations: Strength and Stability

**Figure 1:**
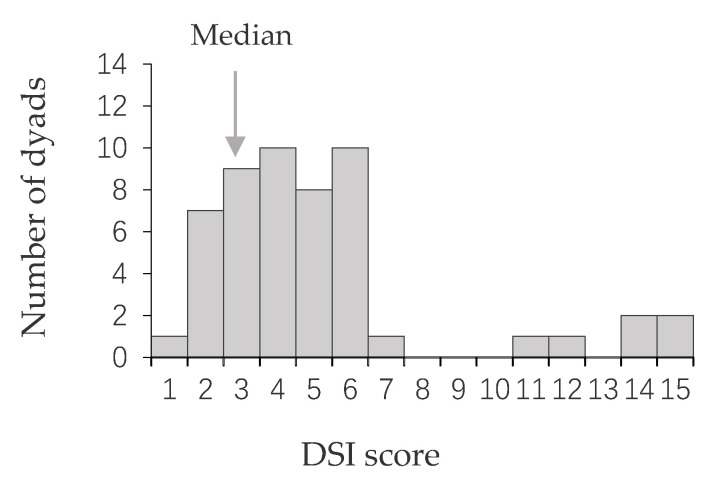
The distribution of male-male DSI scores based on frequency and duration of proximity between all dyads. The DSI indicated the strength of male-male social bonds (mean = 3.8 and median = 3.2).

**Figure 2:**
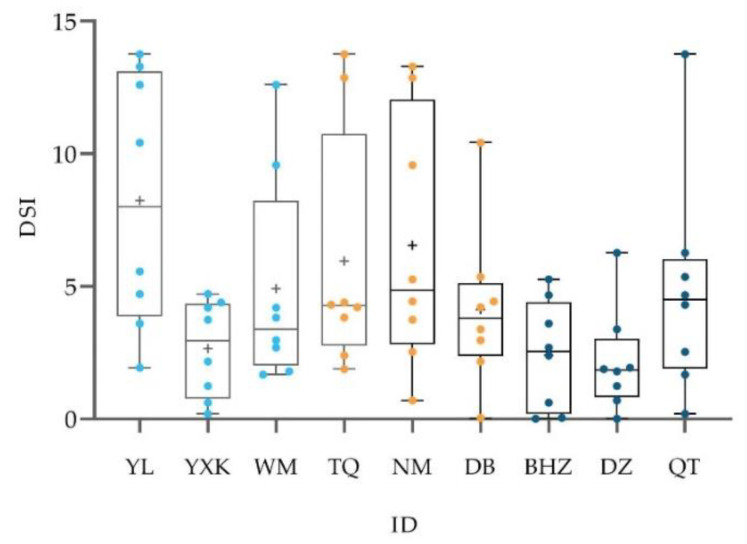
The distribution of DSI scores of males in the group. The plus sign indicates the average DSI score, and different colors indicate the rank class of the males (from left to right: high, middle, and low).

We calculated 55 DSI scores and found that the social relationships among males were highly differentiated, as shown in Figure 1. More than half (63.7%) of the male-male DSI scores were lower than the average. The strength of the social relationship between each male and other individuals in the group was shown in Figure 2.

We recorded 93 incidents of male agonistic support. The DSI score was significantly correlated with the times of coalition (Spearman’s correlation coefficient: t = 4.643, *p* < 0.001). Additionally, bystanders ignored and avoided a male’s request for support 8 times.

### 3.2. Coalitionary Behavior

Model 1: Allies’ choice and social ranking/DSI score

According to model 1, we found that the rank difference between the two attackers played no role in the choice of allies by the supporters. Therefore, we analyzed a simplified model without the interaction terms to elucidate the main factors that influence the supporters’ choice of allies. The social rank and DSI intimacy independently influenced the supporters’ choice of allies, indicating that supporters were more likely to form coalitions with affiliated individuals (DSI score), and potential supporters chose high-ranking individuals as allies during a conflict (Table 4).

Model 2: The joining of supporters and social bond, residence time, and age

We recorded 65 events of coalition behaviors of adult males. According to the results of Model 2 (Table 5), the males who were a part of this group for a longer time had a closer cooperative relationship than the new individuals, and age did not significantly influence the male coalition.

Model 3: Decision-making of partner choice in coalitions 

**Figure 3:**
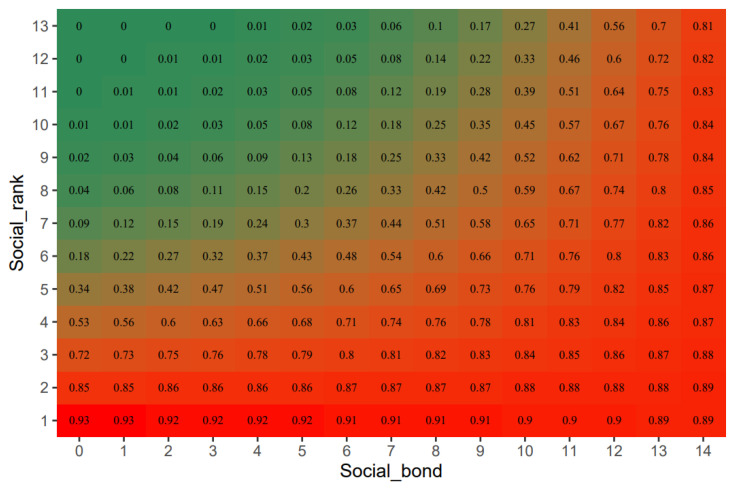
A heat map of member scores for the interaction between social rank and social bond. The values in the heat map represent the probability of coalition success: the closer the color is to red (the heat map score is up to 1), the greater the possibility of a successful coalition, and the closer is to green (the lowest scores is 0), the greater the possibility of failure.

We used logistic regression to fit the interaction between social rank and social bond. The model predicted that supporters were more likely to support individuals with the highest social bond strength and the highest social rank and that the score was a linear combination of their rank, social bond strength, and mutual effect (the interaction of social bonds and social rank on coalitions) (Estimate = 0.054, SE = 0.033, *z* = 1.650, *p* = 0.099). Figure 3 shows that if the recruiter was at the top of the rank (high-ranking), the predicted effect of the social bond of supporters and recruiters on their scores was very slight. As the rank of the recruiter dropped, the influence of social bonds on the success of coalitions became increasingly significant.

## 4. Discussion

In this study, we tested the influence of two factors, social rank and social bond, on the male’s choice of allies, and discussed the influence of social relationship, residence time and age on the coalition. We found that in Tibetan macaques, the male-male social relationship was strong, and the coalition behaviors were obvious. From the perspective of the supporter, supporters were more likely to choose the part with a strong social relationship with them as an ally in the conflict, and the social rank of both attackers also affected the supporters’ choice. The results support predictions 1 and 2. Age had no significant effect on the male coalition, which did not support prediction 3. In addition, the residence time had an influence on the coalition, but the males who stayed for a long time formed more coalitions, which was also different from prediction 4. These results showed that the male Tibetan macaques formed a strong and stable social relationship, and the strength of social bond/rank promotes the formation of the coalition, which was similar to the study of chimpanzees [29].

Affiliative relationships of macaques were stable during the mating season in our study. Social bonds and coalition frequency are often correlated [30]. Our results showed that the higher the social bond strength of the males, the more times they participated in agonistic support. In chimpanzees, forming and maintaining strong social bonds can lead over time to adaptive benefits [29,31]. In zebra finches (*Taeniopygia guttata*), high levels of cooperation were maintained in an iterated Prisoner’s Dilemma game only when interacting with their social partner [32]. Thus, supporters were more likely to choose male allies with whom they had a strong social relationship in agonistic support events. 

Our research showed that social rank had an influence on coalitions, and males were more likely to choose high-ranking allies. The tendency to form coalitions with high-ranking males has been demonstrated repeatedly [33]. During conflicts, the higher the rank of a male, the more likely the supporters were to ally [8]. The supporter would preferentially support individuals with higher rank, and the effects of social bonds on decision of coalition solicitations were independent of the effects of dominance rank [34]. Males prefer high-ranking members since compatible and reliable coalition partners could gain and maintain social rank and enhance reproductive success for themselves [35].

In Tibetan macaques, coalition formation seemed to be based on two criteria (social rank and social bond strength). We use multiple logistic regression to model decision rules in which individuals combine different types of social information. The results revealed that the partner choice in coalitions is related to social relationships and rank. Males choose the coalition that works best for them according to different situations [36,37,38]. In semi-free-ranging Barbary macaques, males are more likely to support maternal relatives than non-relatives, and kinship plays an important role in coalition decision-making [39]. In spotted hyaenas (*Crocuta Crocuta*), social rank and social bonds have been shown to be important in soliciting help or predicting competitor’s supporters [40]. These results are consistent with the view that males choose allies based on different criteria.

## 5. Conclusions

In conclusion, social bonds and social rank both influence the formation of coalitions in Tibetan macaques. Our results suggest that males are more likely to select allies who have a high rank and strong social bond to reduce the risk of ally defecting and increase the success rate of attack. Therefore, males choose different coalition strategies based on the competitive environment to increase coalition advantages. The study of male coalition behavior in multi-male and multi-female societies provides a new perspective and theoretical support for understanding the diversity of primate social behavior and the evolution of social structure. Further research on more social behaviors between males is needed to better understand the evolution of male coalition in primates.

## Figures and Tables

**Table 1:** Definitions of behaviors in Tibetan macaques.

Behavior	Description
Proximity	Two or more individuals are sitting or lying within 5 m of one another.
Stare	An individual looks directly at another individual with its eyes wide open and with its shoulders raised for about 3–5 s.
Ground slap	One individual support with one hand on the ground, the other flaps the ground, and stares at another individual, then it looks down.
Chase	An individual stares at the recipient and rushes at him/her at great speed. The recipient typically flees.
Seizing	One individual grabs the hair of the body, face, or neck of another one, or it may simply grab its ears. Shake it back and forth a few times before letting go.
Bite	The performer grabs the receiver tightly, preventing him/her from fleeing, and bites the recipient vigorously.
Avoid	The performer turns its body away from the attacker as if preparing to flee while displaying a ‘horrified’ facial expression towards the attacker.
Flee	The target of an attack will run in the opposite direction from the attacker.
Agonistic support	The behavior of a social animal in which an individual participates in an ongoing struggle to support one party and attack the other.

**Table 2:** Social ranks of adult male Tibetan macaques.

ID	Social Rank (DS Value)	Age Class *	Residence Time (Year)
YL	1 (22.63)	1	0.4
YXK	2 (22.52)	1	9
WM	3 (14.08)	3	2
TQ	4 (4.90)	2	2
NM	5 (2.90)	2	0.4
DB	6 (−1.20)	1	0.4
BHZ	7 (−17.17)	2	0.3
DZ	8 (−20.08)	1	0.2
QT	9 (−29.02)	1	0.4

* Age class 1: young adult, male ≥7~10 years old; Age class 2: middle-aged, male ≥10~15 years old; Age class 3: old, male ≥15 years old.

**Table 3:** Structure of models 1–3, study on male Tibetan macaques.

Model	Predictive Variables	Response Variables
Model 1	DSI score	A male from individuals in conflict was selected as a coalition partner (Yes/No)
	High rank (Yes/No)	
	The rank difference of individuals in conflict	
Model 2	DSI score of dyad I-J	Number of coalitions of dyad I-J
	The residence time of J	
	Age class of J	
Model 3	The interaction between social rank and social bond	The recruiter is selected as a coalition partner (Yes/No)

**Table 4:** The results of the GLMM to determine the factors that affect agonistic support of Tibetan macaques.

Predictors	Estimate	SE	*Z* Value	*p* Values
DSI score	0.19	0.09	2.02	*p* = 0.044
High rank (Yes)	4.62	0.76	6.06	*p* < 0.001

**Table 5:** The results of the GLMM to determine the factors that affect the coalition of Tibetan macaques.

Predictors	Estimate	SE	*Z* Values	*p* Values
DSI	0.145	0.027	5.223	*p* < 0.001
Residence time	0.119	0.044	2.710	*p* = 0.007
Age class	0.051	0.181	0.287	*p* = 0.774

## Data Availability

Data are available on request.

## References

[1] Radespiel U., Ehresmann P., Zimmermann E. (2001). Contest versus scramble competition for mates: The composition and spatial structure of a population of gray mouse lemurs (*Microcebus murinus*) in North-west Madagascar. Primates.

[2] Cant M.A., Otali E., Mwanguhya F. (2002). Fighting and Mating Between Groups in a Cooperatively Breeding Mammal, the Banded Mongoose. Ethology.

[3] Winkelmann K., Genner M.J., Takahashi T., Ruber L. (2014). Competition-driven speciation in cichlid fish. Nat. Commun..

[4] Luehrs M.-L., Kappeler P.M. (2014). Polyandrous mating in treetops: How male competition and female choice interact to determine an unusual carnivore mating system. Behav. Ecol. Sociobiol..

[5] Girard-Buttoz C., Heistermann M., Rahmi E., Agil M., Fauzan P.A., Engelhardt A. (2014). Costs of and Investment in Mate-Guarding in Wild Long-Tailed Macaques (*Macaca fascicularis*): Influences of Female Characteristics and Male-Female Social Bonds. Int. J. Primatol..

[6] Cassidy K.A., McIntyre R.T. (2016). Do gray wolves (*Canis lupus*) support pack mates during aggressive inter-pack interactions?. Anim. Cogn..

[7] Port M., Schulke O., Ostner J. (2018). Reproductive tolerance in male primates: Old paradigms and new evidence. Evol. Anthropol..

[8] Young C., Schülke O., Ostner J. (2014). How males form coalitions against group rivals and the Pandit/van Schaik coalition model. Behaviour.

[9] Ostner J., Schuelke O. (2014). The evolution of social bonds in primate males. Behaviour.

[10] Hinde R.A. (1983). Primate Social Relationships: An Integrated Approach.

[11] Berghaenel A., Ostner J., Schroeder U., Schuelke O. (2011). Social bonds predict future cooperation in male Barbary macaques, *Macaca sylvanus*. Anim. Behav..

[12] Sandel A.A., Reddy R.B., Mitani J.C. (2017). Adolescent male chimpanzees do not form a dominance hierarchy with their peers. Primates.

[13] Kawazoe T., Sosa S. (2019). Social networks predict immigration success in wild Japanese macaques. Primates.

[14] Duffy K.G., Wrangham R.W., Silk J.B. (2007). Male chimpanzees exchange political support for mating opportunities. Curr. Biol..

[15] Schuelke O., Bhagavatula J., Vigilant L., Ostner J. (2010). Social Bonds Enhance Reproductive Success in Male Macaques. Curr. Biol..

[16] De Moor D., Roos C., Ostner J., Schulke O. (2020). Bonds of bros and brothers: Kinship and social bonding in postdispersal male macaques. Mol. Ecol..

[17] Koykka C., Wild G. (2017). Concessions, lifetime fitness consequences, and the evolution of coalitionary behavior. Mol. Ecol..

[18] Broom M., Koenig A., Borries C. (2009). Variation in dominance hierarchies among group-living animals: Modeling stability and the likelihood of coalitions. Mol. Ecol..

[19] Reddy R.B., Mitani J.C. (2020). Adolescent and young adult male chimpanzees form affiliative, yet aggressive, relationships with females. J. Hum. Evol..

[20] Schoof V.A.M., Jack K.M. (2014). Male social bonds: Strength and quality among co-resident white-faced capuchin monkeys (*Cebus capucinus*). Behaviour.

[21] Altmann J. (1974). Observational study of behavior: Sampling methods. Behaviour.

[22] Berman C.M., Ionica C.S., Li J.H. (2004). Dominance style among Macaca thibetana on Mt. Huangshan, China. Int. J. Primatol..

[23] Li J.H. (1999). The Tibetan Macaque Society: A Field Study.

[24] Gammell M.P., De Vries H., Jennings D.J., Carlin C.M., Hayden T.J. (2003). David’s score: A more appropriate dominance ranking method than Clutton-Brock et al.’s index. Anim. Behav..

[25] Miss F.M., Sadoughi B., Meunier H., Burkart J.M. (2022). Individual differences in co-representation in three monkey species (*Callithrix jacchus, Sapajus apella* and *Macaca tonkeana*) in the joint Simon task: The role of social factors and inhibitory control. Anim. Cogn..

[26] Silk J., Cheney D., Seyfarth R. (2013). A Practical Guide to the Study of Social Relationships. Evol. Anthropol..

[27] Bissonnette A., de Vries H., van Schaik C.P. (2009). Coalitions in male Barbary macaques, *Macaca sylvanus*: Strength, success and rules of thumb. Anim. Behav..

[28] Bates D., Mächler M., Bolker B., Walker S. (2015). Fitting Linear Mixed-Effects Models Using lme4. J. Stat. Softw..

[29] Bray J., Feldblum J.T., Gilby I.C. (2021). Social bonds predict dominance trajectories in adult male chimpanzees. Anim. Behav..

[30] Gilby I.C., Brent L.J.N., Wroblewski E.E., Rudicell R.S., Hahn B.H., Goodall J., Pusey A.E. (2013). Fitness benefits of coalitionary aggression in male chimpanzees. Mol. Ecol. Sociobiol..

[31] Bray J., Gilby I.C. (2020). Social relationships among adult male chimpanzees (*Pan troglodytes schweinfurthii*): Variation in the strength and quality of social bonds. Mol. Ecol. Sociobiol..

[32] St-Pierre A., Larose K., Dubois F. (2009). Long-term social bonds promote cooperation in the iterated Prisoner’s Dilemma. Proc. R. Soc. B-Biol. Sci..

[33] Chapais B. (1995). Alliances as means of competition in primates: Evolutionary, developmental and cognitive aspects. Yearb. Phys. Anthropol..

[34] Perry S., Barrett H.C., Manson J.H. (2004). White-faced capuchin monkeys show triadic awareness in their choice of allies. Anim. Behav..

[35] Neumann C., Kulik L., Agil M., Engelhardt A., Widdig A. (2022). Temporal dynamics and fitness consequences of coalition formation in male primates. Proc. R. Soc. B Biol. Sci..

[36] Carne C., Wiper S., Semple S. (2011). Reciprocation and interchange of grooming, agonistic support, feeding tolerance, and aggression in semi-free-ranging Barbary macaques. Am. J. Primatol..

[37] Gerber L., Connor R.C., King S.L., Allen S.J., Wittwer S., Bizzozzero M.R., Friedman W.R., Kalberer S., Sherwin W.B., Wild S. (2020). Affiliation history and age similarity predict alliance formation in adult male bottlenose dolphins. Mol. Ecol..

[38] Gerber L., Wittwer S., Allen S.J., Holmes K.G., King S.L., Sherwin W.B., Wild S., Willems E.P., Connor R.C., Krutzen M. (2021). Cooperative partner choice in multi-level male dolphin alliances. Sci. Rep..

[39] Widdig A., Streich W.J., Tembrock G. (2000). Coalition formation among male Barbary macaques (*Macaca sylvanus*). Am. J. Primatol..

[40] Engh A.L., Siebert E.R., Greenberg D.A., Holekamp K.E. (2005). Patterns of alliance formation and postconflict aggression indicate spotted hyaenas recognize third-party relationships. Anim. Behav..

